# Pseudoscorpiones and Scorpiones of Canada

**DOI:** 10.3897/zookeys.819.27121

**Published:** 2019-01-24

**Authors:** Elyssa Cameron, Christopher M. Buddle

**Affiliations:** 1 Department of Natural Resource Sciences, Faculty of Agricultural and Environmental Sciences, McGill University, Ste-Anne-de-Bellevue, Quebec, H9X 3V9, Canada McGill University Montreal Canada

**Keywords:** Arachnida, biodiversity assessment, Biota of Canada, Pseudoscorpiones, Scorpiones

## Abstract

Twenty-five species of pseudoscorpions are known from Canada, a five-fold increase since an assessment from 1979. The diversity and distribution of Canadian species are poorly known and at least 27 more species are expected to be found in the country. Currently 46 Barcode Index Numbers are assigned to Canadian specimens, suggesting a high level of undocumented diversity. Only one scorpion species is known from Canada and no other species are expected.

## Pseudoscorpiones

Pseudoscorpions are a group of small (i.e., typically less than 5 mm in length), carnivorous arachnids that have a cryptic nature and are therefore not frequently observed. Thus, pseudoscorpions remain undersampled and understudied globally (Harvey 2002). Harvey’s (1990) catalogue was a major contribution for this taxon, and the first cladistics tests of the group, outlining two suborders, 24 families, 425 genera, and 3200 species worldwide, was done by Harvey soon after (Harvey 1992). More recent phylogenetic analyses of pseudoscopions were completed by Murienne et al. (2008). There are estimated to be over 500 species in North America (Harvey 2013). The online Pseudoscopions of the World catalogue is the updated and comprehensive information source about pseudoscorpions (Harvey 2013).

Little is known about pseudoscorpion distribution, ecology, and taxonomy in Canada (Buddle 2005); however, some advances in knowledge of the Canadian fauna have been made since the group was reviewed by Dondale (1979). Some examples of research on pseudoscorpions in Canada include Koponen’s (1994) assessment on the diversity and abundance of this group in Quebec peatlands, work by Koponen and Sharkey (1988) on northern records for some species, taxonomic work by Muchmore (1990, 1996), and ecological research by Buddle (2015). Buddle (2005) published a pseudoscorpion primer, which gives general information about ecology, morphology, collection, status, and distribution, and cites additional key literature about the taxon in Canada. Buddle (2010) published the first photographic key to family and genus for the pseudoscorpions of Canada and adjacent contiguous states of the United States of America.

Dondale (1979) surveyed literature and reported five species of pseudoscorpions from Canada, but he did not report family affiliations. At present, 25 species of pseudoscorpions from nine families occur in Canada, a five-fold increase in species since 1979 (Table 1). Chernetidae presently has the highest species richness in Canada, with eight known species, followed by the families Cheliferidae and Chthoniidae with four each. At least four species are known to be introduced to North America, one of which occurs in Canada, the cosmopolitan *Chelifercancroides* (L.) (Cheliferidae) typically associated with human dwellings. Two other non-native species, *Roncuslubricus* L. Koch (Neobisiidae) and *Cheiridiummuseorum* (Leach) (Cheiridiidae), may occur in Canada.

In total, 46 Barcode Index Numbers (BINs) (Ratnasingham and Herbert 2013) have been assigned to Canadian pseudoscorpion specimens (Table 1). While this method of delineating Operational Taxonomic Units must be taken with caution with pseudoscorpions (Arabi et al. 2012), it remains one way to estimate broader taxonomic diversity within this taxon. The families with the most BINs represented are Neobisiidae (18), Chthoniidae (11), and Chernetidae (10) (Table 1). As the number of BINs is almost double the number of documented species, it is possible that many more species remain to be discovered or described in Canada. To estimate the number of undocumented species for each family, we considered BIN data as well as the presence of species in adjacent states of the USA that have not yet been recorded in Canada but are likely to be there. We also recognize that there may be Holarctic species yet to be discovered. Based on these information sources, we estimate at least 27 additional species will be documented in the country, many from the family Chernetidae (Table 1). As an example, although not yet known from Canada, we expect the family Garypinidae to eventually be found given the proximity to localities in the USA. Neobisiidae, and to a lesser extent Chthoniidae, show many more BINs than known species in Canada (Table 1), perhaps suggesting problems with this method for pseudoscorpions (e.g., Arabi et al. 2012). Other possible explanations include: undersampling, the indication that more species are present among collected material than recognized (e.g., cryptic species), and that there may be multiple BINs for a single species. More generally, the discrepancy indicates that these families could be the foci for careful and increased sampling and integrated taxonomic research. As such, estimates of expected number of species for these families are certainly conservative.

Dondale (1979) noted the order as being ‘transcontinental’ and, although distributions are still poorly known in Canada, the group as a whole is certainly transcontinental with a deep evolutionary history in North America. However, the ‘ecological’ distribution, and sampling of pseudoscorpions in North America, should indeed reveal that they are found in virtually all habitable space on the continent, perhaps other than the high Arctic. Individuals have been recorded from British Colombia to the Maritimes and Newfoundland as well as from the southern border to 67° N in the Yukon (Buddle 2015). Pseudoscorpions have been recorded in all ecozones, but data deficiency is a problem as known distributions are based on few published records. Therefore, the distributions included in Table 1 are incomplete for most (if not all) families for many reasons, including lack of interest and undersampling. Chernetidae and Neobisiidae are found as far north as the Taiga Plains ecozone. Others, such as Chthoniidae, are mostly recorded from southern ecozones where they are widespread, and survey work in caves may reveal even more species across a range of latitudes.

Although the composition and distribution of the Canadian fauna is better known now than it was in 1979, there are still enormous gaps in our knowledge. Due to their cryptic nature, and sometimes clumped distribution in a habitat, there are seldom systematic collections of pseudoscorpions, and museums have specimens waiting to be sorted and identified. Nonetheless, without directed sampling, especially in some ecozones and habitats, it will be a long time before the faunal composition and distribution is adequately known. As pseudoscorpions are encountered frequently in pitfall traps, Berlese and Tullgren extractions of litter and soil, during rearing from dead wood, in nests, caves, and phoretic on other animals, there are many opportunities to preserve such material for future study, thereby contributing to the body of knowledge specimen by specimen. Moreover, there is a large gap in knowledge of fundamental natural history of pseudoscorpions from Canada, although the foundation for ecological work which was well established by Muchmore (1973) and Weygoldt (1969) remains an important source. There are plentiful opportunities to learn about the ecology of these animals, including what they eat, how they live, and the relationships between pseudoscorpions and other animals (e.g., phoresy). Fundamentally, we need the curious mind to pay attention to these small and marvellous creatures.

**Table 1.** Census of Pseudoscorpiones and Scorpiones in Canada. Information sources are Buddle (2005, 2010).

Taxon^1^	No. species reported in Dondale (1979)^2^	No. species currently known from Canada^3^	No. BINs^4^ available for Canadian species	Est. no. undescribed or unrecorded species in Canada	General distribution by ecozone^5^
**Order Pseudoscorpiones**					
Cheiridiidae	?	1	1	0	Prairies, possibly Boreal Plains and others
Cheliferidae	?	4 (1)	3	3	most southern ecozones
Chernetidae	?	8	10	11–15	Taiga Plains, most southern ecozones
Chthoniidae	?	4	11	4	widespread, all southern ecozones
Garypinidae	0	0	0	1	Pacific Maritime, Montane Cordillera?
Larcidae	?	1	0	1	Prairies, Mixedwood Plains, possibly others
Neobisiidae	?	3	18	3	Taiga Plains, Boreal Plains, Boreal Shield, Newfoundland Boreal, Mixedwood Plains
Pseudogarypidae	?	1	0	0	Mixedwood Plains, Atlantic Maritime, possibly others
Syarinidae	?	3	3	0	Mixedwood Plains, Boreal Shield, Boreal Plains, possibly others
**Total**	**5**	**25 (1)**	**46**	**27**	
**Order Scorpiones**					
Vaejovidae	1	1	1	0	Prairies, Western Interior Basin

^1^Classification follows that of Harvey (1992). ^2^Dondale (1979) did not provide family affiliations. ^3^Numbers in parentheses denote numbers of non-native species included in the totals. ^4^Barcode Index Number, as defined in Ratnasingham and Hebert (2013). ^5^See figure 1 in Langor (2019) for a map of ecozones.

## Scorpiones

The northern scorpion, *Paruroctonusboreus* (Girard), is the only species of scorpion found in Canada, as reported by Dondale (1979). The species is found in the southern Prairies ecozone in Alberta and Saskatchewan and in the Western Interior Basin ecozone, specifically in the southern Okanagan Valley of British Colombia (Johnson 2004). No other species of scorpion is thought to live in Canada. Canadian specimens of this species have not yet been DNA barcoded.

In southern Alberta, the northern scorpion inhabits dry, eroded riverbed slopes and lives in rock fissures or narrow cavities and emerges at night to hunt (Johnson 2004). The species is not efficiently caught using pitfall traps and is best observed by flipping over stones or dried cattle dung (C Sheffield pers. comm.) in the daytime or with the use of ultraviolet lights at night (Johnson 2004).
